# Assessment of TG/HDL-C and METS-IR as markers for diabetic retinopathy risk and severity in type 2 diabetes

**DOI:** 10.3389/fmed.2026.1767310

**Published:** 2026-04-10

**Authors:** Qiuhui Tian, Yinyin Zhang, Qiumei Cao, Yu Liu, Xuemei Ou, Zerun Zeng, Lili Xu

**Affiliations:** 1 Department of Gastroenterology, Jiangjin Hospital, Chongqing University of Chinese Medicine, Chongqing, China; 2 Department of Gastroenterology, Chongqing Jiangjin District Hospital of Chinese Medicine, Chongqing, China

**Keywords:** diabetic retinopathy, metabolic score for insulin resistance, proliferative diabetic retinopathy, triglyceride-to-high-density lipoprotein cholesterol ratio, type 2 diabetes mellitus

## Abstract

**Objective:**

To investigate the association of triglyceride-to-high-density lipoprotein cholesterol (TG/HDL-C) and metabolic score for insulin resistance (METS-IR) with diabetic retinopathy (DR) in patients with type 2 diabetes (T2DM), to identify factors independently associated with DR, and to explore their relationship with DR severity.

**Methods:**

This study enrolled 329 hospitalized patients with T2DM (75 with DR, 254 without). LASSO regression was applied to select factors associated with DR, followed by multivariable logistic regression. Model performance was assessed using area under the ROC curve (AUC), bootstrap internal validation (1,000 resamples) for optimism correction, calibration plots, and decision curve analysis (DCA). For the exploratory analysis of proliferative diabetic retinopathy (PDR, *n* = 32), Firth’s penalized logistic regression was used to identify associated factors.

**Results:**

LASSO identified age, fasting C-peptide, TG/HDL-C and METS-IR as factors associated with DR. Multivariable logistic regression confirmed that age (OR = 1.05 per year, 95% CI 1.02–1.08), TG/HDL-C (OR = 1.58 per unit, 1.33–1.88) and METS-IR (OR = 1.08 per unit, 1.01–1.15) were positively associated with DR, whereas fasting C-peptide showed an inverse association (OR = 0.11 per ng/mL, 0.04–0.29). The model achieved an AUC of 0.934 (95% CI 0.905–0.963), with an optimism-corrected C-index of 0.929 (0.887–0.971) after bootstrap validation. Calibration was satisfactory (intercept = 0.00, slope = 1.00, Brier score = 0.08), and DCA indicated positive net benefit within the 5–30% threshold range. Exploratory analysis of PDR using Firth’s penalized regression showed no statistically significant association for TG/HDL-C (OR = 3.10, 1.77–7.21, *p* < 0.001), METS-IR (OR = 1.80, 1.31–3.07), likely due to limited sample size.

**Conclusion:**

TG/HDL-C and METS-IR are independently associated with DR in hospitalized patients with T2DM. The model incorporating these factors demonstrates good discrimination and calibration, with potential utility for risk stratification. Exploratory analysis of PDR, constrained by small sample size, did not confirm significant associations, underscoring the need for larger studies on progression to proliferative disease.

## 1 Introduction

According to data from the International Diabetes Federation (IDF), the global prevalence of diabetes among adults aged 20–79 years was approximately 10.5% in 2021, corresponding to ~536.6 million affected individuals. This places a substantial burden on global health systems, with diabetes-related healthcare expenditures totaling approximately $966 billion. It is projected that the global number of people living with diabetes will rise to 783.2 million by 2045 (1). Among the diverse complications of diabetes, diabetic retinopathy (DR) is one of the most prevalent microvascular complications worldwide, with an overall pooled prevalence of ~22.27% (2). Notably, DR has emerged as a leading cause of vision impairment and blindness in the working-age population (18–64 years) (3).

Notably, more than 50% of global cases of blindness or vision impairment attributed to DR are distributed across the Asia-Pacific region (4). A multicenter prognostic study demonstrated that the median time for progression from non-proliferative diabetic retinopathy (NPDR) or diabetic macular edema (DME) to proliferative diabetic retinopathy (PDR) in patients with type 2 diabetes mellitus (T2DM) is approximately 1.89 years (5). A 2019 systematic review further reported that the annual progression rate from early-stage DR to vision-threatening DR ranges from 3.4 to 12.3% (6). Beyond impairing visual function, DR also significantly elevates the risk of cardiovascular events and all-cause mortality in affected patients (7).

Early diagnosis and timely intervention can effectively delay the progression of DR and reduce the risk of irreversible blindness (8). However, early-stage DR lesions are typically asymptomatic, making it difficult for patients to detect the condition independently. The American Academy of Ophthalmology (AAO) recommends that patients with T2DM undergo fundus screening at the time of T2DM diagnosis, followed by regular annual follow-up examinations thereafter. Although dilated fundus examination is widely recognized as the gold standard for DR screening, approximately 40% of eligible patients fail to complete annual screenings in clinical practice (9). This screening gap is largely attributed to the technical complexity of the procedure, as well as transient adverse effects following pupil dilation—including temporary visual impairment, photophobia, and ocular discomfort—which significantly compromise patient adherence to screening protocols.

Existing studies have identified multiple risk factors associated with the development of DR, including diabetes duration, long-term hyperglycemia, hypertension, dyslipidemia (10), diabetic kidney disease (DKD) (11), pregnancy (12), and abdominal obesity (13). However, most prior research has primarily focused on the “onset” of DR, whereas the exploration of predictive factors for DR “progression”—particularly progression to vision-threatening stages such as PDR—remains inadequate, and the findings from existing studies are inconsistent. The present study aims to investigate the associations of the triglyceride-to-high-density lipoprotein cholesterol (TG/HDL-C) and the metabolic score for insulin resistance (METS-IR) with DR in patients with T2DM, aiming to identify factors independently associated with DR and to assess their relationship with disease severity.

## 2 Method

### 2.1 Study design and populations

This cross-sectional study enrolled 420 patients with T2DM who were admitted to the Department of Endocrinology, Chongqing Jiangjin District Hospital of Chinese Medicine, between January 2024 and May 2025. Eligible participants had a documented T2DM duration of 5–10 years and were receiving intensive insulin therapy. All clinical data, laboratory test results, and retinal lesion conditions of all patients should be based on the initial examination records made at the time of admission. Inclusion criteria were as follows: All enrolled patients were older than 18 years, had been diagnosed with T2DM according to the Chinese Guideline for the Prevention and Treatment of Type 2 Diabetes Mellitus (2020 edition), and had completed a comprehensive fundus examination. Exclusion criteria included: (1) T2DM duration < 5 years or > 10 years; (2) diagnosis of other diabetes subtypes (e.g., type 1 diabetes, gestational diabetes mellitus, monogenic diabetes, or latent autoimmune diabetes in adults); (3) presence of severe non-diabetic ocular disorders (e.g., retinal vein occlusion, age-related macular degeneration, primary open-angle glaucoma, or high myopic retinopathy); (4) comorbidity with severe chronic diseases, such as decompensated heart failure, cirrhosis, end-stage renal disease, acute cerebrovascular events, hematologic malignancies, or solid tumors; (5) history of ocular surgery (e.g., cataract extraction, vitrectomy) or retinal laser photocoagulation therapy; (6) use of lipid-lowering medications (e.g., statins or fibrates) within the preceding 3 months; (7) incomplete clinical data that precluded accurate patient grouping or statistical analysis. The study protocol was approved by the Ethics Committee of the corresponding author’s affiliated institution (Approval No: ZYY2025015). Written informed consent was obtained from all participants prior to study enrollment.

### 2.2 Sample size calculation

The final multivariable model included four predictors. According to the criterion proposed by Peduzzi (14),that the event number per variable (EPV) is ≥ 10, 75 DR events could support a stable estimation of up to 7–8 variables (EPV = 18.75). Further, using the sample size framework proposed by Riley (15), under the condition of expected Cox-Snell R^2^ = 0.15 and event rate of 22.8%, 75 events met the shrinkage factor > 0.9 and prediction accuracy requirements, with a relatively low risk of overfitting.

### 2.3 DR grading standards

DR was graded through dilated fundus examination according to the international standards set forth in AAO Diabetic Retinopathy Preferred Practice Pattern (2019) (16). The grading criteria were as follows: (1) No clinically significant DR: no abnormalities detected; (2) NPDR, categorized as mild (microaneurysms only), moderate (microaneurysms plus other findings less severe than severe NPDR), or severe NPDR. Severe NPDR was defined by either the AAO “4–2-1” rule, requiring at least one of the following in the absence of PDR: severe intraretinal hemorrhages/microaneurysms in one or more quadrants, venous beading in two or more quadrants, or moderate intraretinal microvascular abnormalities (IRMA) in one or more quadrants; or by international criteria: more than 20 intraretinal hemorrhages in each of all four quadrants, venous beading in two or more quadrants, or prominent IRMA in one or more quadrants, without progression to PDR; (3) PDR: presence of neovascularization (of the disc or elsewhere), vitreous hemorrhage, or preretinal hemorrhage. Fundus images were independently graded by two ophthalmologists (associate chief physician or above), both masked to patients’ clinical data. Grading was based solely on fundus findings. Disagreements were resolved by consensus or, if consensus could not be reached, adjudicated by a third senior ophthalmologist specializing in retinal diseases.

### 2.4 Data collection

Demographic and laboratory data were extracted from the electronic medical record (EMR) system within 24 h of patient admission. Collected variables included general information (age, sex, height, weight, smoking history, drinking history, systolic and diastolic blood pressure at the time of admission) and laboratory indicators: hematological parameters (white blood cell count [WBC], red blood cell count [RBC], hemoglobin [HGB], platelet count [PLT], neutrophil count [NEU], lymphocyte count [LYM], monocyte count [MON]), coagulation parameters (prothrombin time [PT], international normalized ratio [INR], activated partial thromboplastin time [APTT], thrombin time [TT], fibrinogen [Fib]), metabolic and glycemic parameters (glycated hemoglobin [HbA1c], fasting plasma glucose [FPG], total cholesterol [TC], triglycerides [TG], high-density lipoprotein cholesterol [HDL-C], low-density lipoprotein cholesterol [LDL-C]), liver and kidney function markers (alanine aminotransferase [ALT], aspartate aminotransferase [AST], total bilirubin [TBIL], direct bilirubin [DBIL], blood urea nitrogen [BUN], serum creatinine [CREA], uric acid [UA]), electrolyte and myocardial enzyme measures (sodium [Na], potassium [K], lactate dehydrogenase [LDH], creatine kinase [CK], creatine kinase-MB [CK-MB]), and a renal injury-related parameter (urinary albumin-to-creatinine ratio [UACR]). In addition, 0-h blood glucose and fasting C-peptide levels from the oral glucose tolerance test (OGTT) were recorded. All patients underwent dilated fundus examination to confirm the presence and severity of DR. Data collection and review were performed independently by two or more authors. Missing items were supplemented by rechecking clinical records; unobtainable data were excluded, and a complete case dataset was used for final analysis.

### 2.5 Calculation of clinical parameters

Body mass index (BMI) = Weight (kg) / height 2 (m^2^);

TG/HDL-C = TG (mmol/L) / HDL-C (mmol/L);

METS-IR = ln [2 × FPG(mg/dL) + TG(mg/dL)] × BMI(kg/m^2^) / ln (HDL-C (mg/dL)) (17).

### 2.6 Grouping criteria

All patients underwent mydriatic fundus examination and were classified into diabetic retinopathy (DR) and non-DR (NDR) groups based on the presence of retinopathy. DR patients were further stratified into non-proliferative (NPDR) and proliferative (PDR) stages according to disease severity.

### 2.7 Statistical analysis

All statistical analyses were performed using R software (version 4.2.2) and SPSS (version 27.0). Normally distributed quantitative data were compared between groups using the independent samples t-test and are presented as mean ± standard deviation; non-normally distributed data were expressed as median (Q1, Q3) and compared using the Mann–Whitney U test. Categorical data were summarized as numbers (percentages) and analyzed with the chi-square test or Fisher’s exact test, as appropriate. Variable selection was performed using LASSO regression. Multicollinearity among selected variables was assessed with variance inflation factors (VIFs); VIF < 5 indicated no concerning collinearity. After excluding variables with significant multicollinearity, binary logistic regression was used to identify factors independently associated with DR. A nomogram was constructed using the rms package in R (v4.2.2). Model discrimination and calibration were evaluated using the ROC curve, calibration plot, and Hosmer–Lemeshow test, with internal validation performed by bootstrap resampling (1,000 iterations). For the analysis of DR severity, binary logistic regression and Firth’s penalized logistic regression were applied to identify factors associated with PDR. All tests were two-sided, with *p* < 0.05 considered statistically significant.

## 3 Results

### 3.1 Baseline characteristics

**Figure 1:**
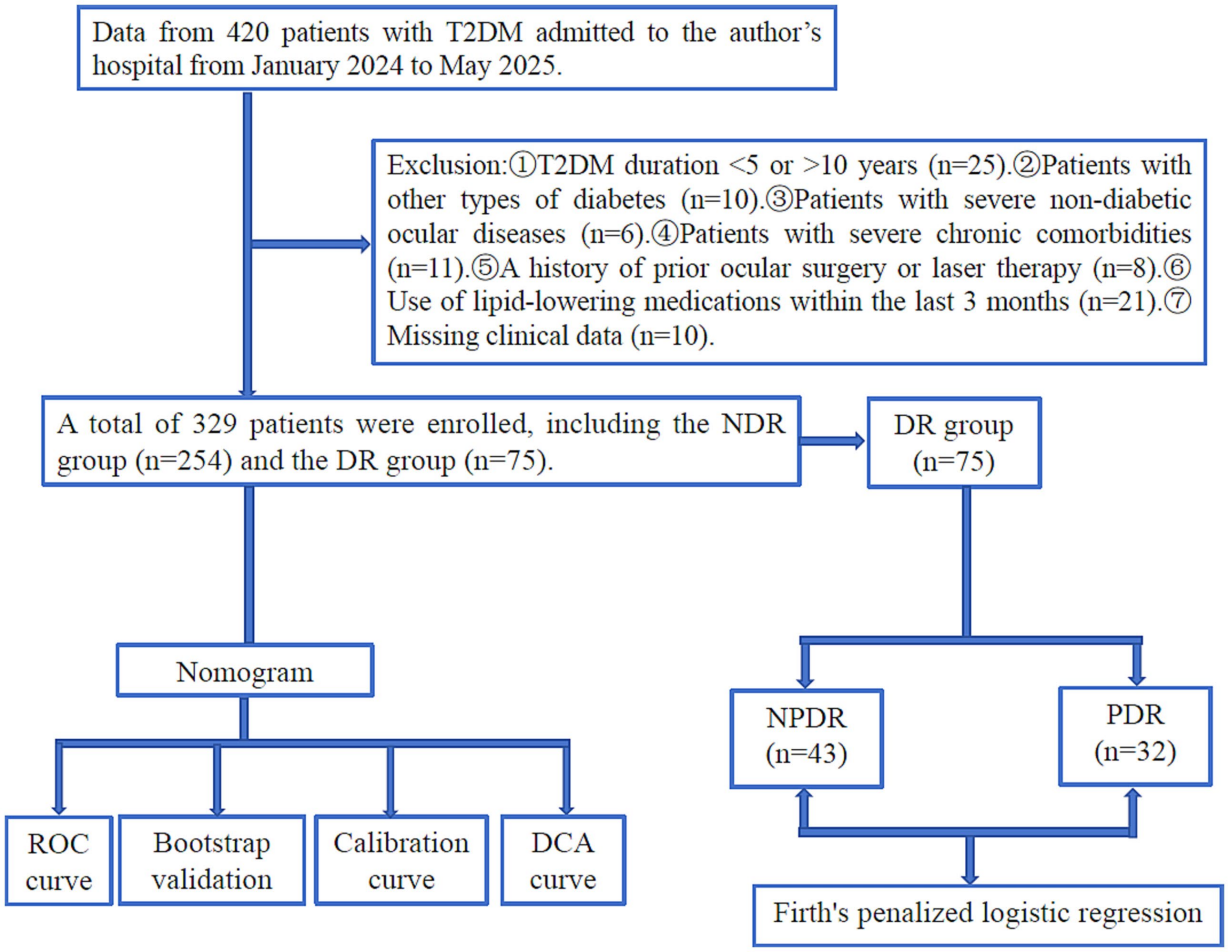
Study flow chart.

A total of 329 patients with T2DM were included in the final analysis (Figure 1) and stratified by DR status. Compared with the NDR group, patients with DR had significantly higher age, BMI, PLT, Fib, Gluc, LDH, TG/HDL-C and METS-IR (*p* < 0.05), and lower RBC, HGB, ALT, AST and fasting C-peptide (*p* < 0.05). No significant differences were observed for other variables (Table 1).

**Table 1:** Comparison of baseline characteristics between the DR and NDR groups.

Characteristic	Overall *N* = 329	DR *N* = 254	NDR *N* = 75	*p*-value
Sex, *n* (%)				0.591
Female	149 (45.3%)	113 (44.5%)	36 (48.0%)	
Male	180 (54.7%)	141 (55.5%)	39 (52.0%)	
Age (year)	58 (51, 66)	57 (50, 66)	60 (56, 67)	0.013
History of smoking				0.856
No	161 (48.9%)	125 (49.2%)	36 (48.0%)	
Yes	142 (43.2%)	108 (42.5%)	34 (45.3%)	
Once upon a time, now quit	26 (7.9%)	21 (8.3%)	5 (6.7%)	
History of drinking				0.562
No	156 (47.4%)	122 (48.0%)	34 (45.3%)	
Yes	148 (45.0%)	111 (43.7%)	37 (49.3%)	
Once upon a time, now quit	25 (7.6%)	21 (8.3%)	4 (5.3%)	
SBP (mmHg)	131 (120, 145)	131 (120, 143)	130 (123, 149)	0.193
DBP (mmHg)	83 (75, 93)	83 (75, 93)	83 (74, 94)	0.815
WBC (×10^9^/L)	6.70 (5.53, 8.22)	6.69 (5.53, 8.20)	6.70 (5.53, 8.63)	0.524
RBC (×10^12^/L)	4.67 (4.27, 5.12)	4.72 (4.38, 5.18)	4.47 (4.10, 4.76)	0.001
HGB (g/L)	141 (131, 154)	143 (133, 156)	137 (125, 145)	<0.001
PLT (×10^9^/L)	204 (171, 252)	199 (168, 250)	222 (187, 259)	0.042
NEU (×10^9^/L)	4.46 (3.40, 5.90)	4.43 (3.30, 5.59)	4.50 (3.55, 6.48)	0.190
LYM (×10^9^/L)	1.60 (1.18, 2.06)	1.70 (1.20, 2.13)	1.50 (1.17, 1.90)	0.059
MON (×10^9^/L)	0.35 (0.27, 0.45)	0.34 (0.27, 0.45)	0.36 (0.26, 0.46)	0.703
PT (Sec)	12.70 (12.30, 13.40)	12.80 (12.30, 13.40)	12.70 (12.20, 13.30)	0.307
INR	1.06 (1.02, 1.11)	1.06 (1.02, 1.11)	1.05 (1.01, 1.10)	0.317
PTA (Sec)	89 (84, 93)	89 (84, 92)	90 (84, 94)	0.307
APTT (Sec)	26.7 (24.2, 28.8)	26.7 (24.2, 28.7)	26.6 (24.2, 29.2)	0.699
TT (Sec)	14.32 (13.45, 15.13)	14.26 (13.45, 15.11)	14.74 (13.39, 15.32)	0.339
Fib (g/L)	2.91 (2.50, 3.53)	2.86 (2.47, 3.46)	3.07 (2.68, 4.14)	0.008
HbA1c (%)	10.70 (8.60, 12.10)	10.80 (8.60, 12.10)	10.30 (8.70, 11.80)	0.595
FPG (mmol/L)	11.9 (9.0, 16.6)	11.5 (8.8, 16.1)	13.5 (10.0, 19.1)	0.023
TC (mmol/L)	5.09 (4.37, 6.10)	5.08 (4.32, 6.09)	5.24 (4.45, 6.11)	0.950
LDL-C (mmol/L)	2.90 (2.34, 3.55)	2.81 (2.32, 3.54)	3.05 (2.40, 3.59)	0.264
ALT (U/L)	20 (14, 34)	21 (14, 36)	17 (14, 21)	0.003
AST (U/L)	20 (16, 28)	21 (17, 29)	18 (16, 24)	0.020
TBIL (μmol/L)	13 (10, 16)	13 (10, 17)	13 (10, 15)	0.087
DBIL (μmol/L)	2.20 (1.70, 2.80)	2.30 (1.70, 2.80)	2.10 (1.70, 2.70)	0.311
BUN (mmol/L)	5.91 (4.82, 7.64)	5.77 (4.82, 7.24)	6.47 (4.83, 8.59)	0.050
CREA (μmol/L)	61 (50, 74)	59 (49, 72)	64 (50, 87)	0.058
UA (μmol/L)	285 (221, 364)	282 (220, 366)	294 (231, 353)	0.564
K (mmol/L)	4.10 (3.85, 4.33)	4.10 (3.85, 4.32)	4.11 (3.85, 4.45)	0.653
Na (mmol/L)	135.5 (133.1, 137.5)	135.5 (132.9, 137.5)	135.6 (133.6, 137.3)	0.878
CK (U/L)	71 (50, 97)	72 (51, 101)	68 (46, 88)	0.108
LDH (U/L)	146 (125, 183)	143 (124, 182)	153 (138, 195)	0.033
CK-MB (U/L)	15 (12, 19)	15 (12, 19)	15 (12, 19)	0.830
UACR (mg/g)	30 (13, 76)	24 (13, 65)	37 (16, 139)	0.109
0-h glucose (mmol/L)	7.82 (6.52, 9.19)	7.87 (6.42, 9.29)	7.82 (6.84, 9.17)	0.558
Fasting C peptide (ng/mL)	1.06 (0.61, 1.85)	1.21 (0.66, 2.20)	0.84 (0.49, 1.09)	<0.001
BMI (kg/m^2^)	24.6 (22.5, 26.7)	24.2 (22.0, 26.6)	25.4 (23.8, 27.0)	0.009
TG/HDL-C	2.2 (1.1, 5.7)	1.8 (0.9, 3.2)	8.4 (4.6, 10.7)	<0.001
METS-IR	35 (31, 39)	34 (29, 38)	39 (37, 42)	<0.001

SBP, systolic blood pressure; DBP, diastolic blood pressure; WBC, white blood cell count; RBC, red blood cell; HGB, hemoglobin; PLT, platelet; NEU, neutrophils; LYM, lymphocytes; MON, monocytes; PT, prothrombin time; INR, international normalized ratio; PTA, prothrombin activity; APTT, activated partial thromboplastin time; TT, thrombin time; Fib, fibrinogen; HbA1c:hemoglobin A1c; FPG, fasting plasma glucose; TC, total cholesterol; LDL-C, low-density lipoprotein Cholesterol; ALT, alanine aminotransferase; AST, aspartate aminotransferase; TBIL, total bilirubin; DBIL, direct Bilirubin; BUN, blood urea nitrogen; CREA, creatinine; UA, uric acid; K:kalium; Na, natrium; CK, creatine Kinase; LDH, lactate dehydrogenase; CK-MB, creatine kinase-myocardial band; UACR, urine albumin-to-creatinine ratio; 0-h glucose:0-h Glucose; BMI, body mass index; TG/HDL-C, triglyceride to high-density lipoprotein cholesterol ratio; METS-IR, metabolic syndrome insulin resistance index.

### 3.2 Factors independently associated with DR

**Figure 2:**
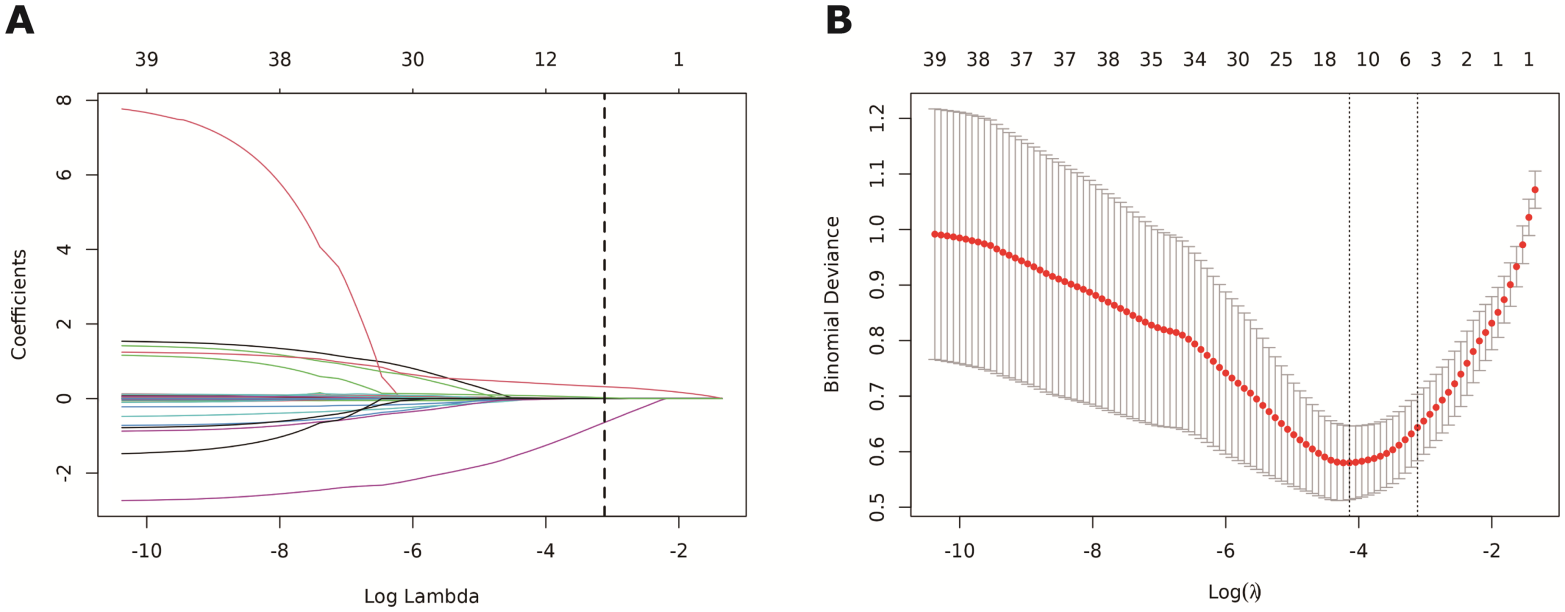
LASSO regression analysis was used to screen the influencing factors of DR in T2DM patients.

LASSO regression selected four predictors with non-zero coefficients from 39 candidate variables: age, fasting C-peptide, TG/HDL-C and METS-IR (Figures 2A,B). All variance inflation factors (VIFs) were <2, indicating no concerning multicollinearity (Table 2). Multivariable logistic regression confirmed that age (OR = 1.05 per year, 95% CI 1.02–1.09), TG/HDL-C (OR = 1.71 per unit, 1.46–2.00) and METS-IR (OR = 1.13 per unit, 1.05–1.21) were positively associated with DR, whereas fasting C-peptide showed an inverse association (OR = 0.12 per ng/mL, 0.05–0.27) (Table 3). The final model was:

**Table 2:** Use variance inflation factors (VIFs) to assess the multicollinearity among significant variables.

Characteristic	VIF	VIF_CI_low	VIF_CI_high	SE_factor	Tolerance
Age (year)	1.21	1.10	1.43	1.10	0.83
Fasting C-peptide (ng/mL)	1.47	1.30	1.72	1.21	0.68
TG/HDL-C	1.55	1.37	1.82	1.25	0.64
METS-IR	1.30	1.17	1.52	1.14	0.77

**Table 3:** Identification of risk factors for DR in patients with T2DM using logistic regression analysis.

Characteristic	Univariable			Multivariable		
	OR	95% CI	*p*-value	OR	95% CI	*p*-value
Age (year)	1.02	1.00, 1.05	0.037	1.05	1.02, 1.09	0.001
Fasting C-peptide (ng/mL)	0.34	0.22, 0.52	<0.001	0.12	0.05, 0.27	<0.001
TG/HDL-C	1.56	1.41, 1.73	<0.001	1.71	1.46, 2.00	<0.001
METS-IR	1.17	1.11, 1.23	<0.001	1.13	1.05, 1.21	<0.001

Log [Pˆ1 − Pˆ] = −8.978 + 0.053 (Age) − 2.129 (Fasting C-peptide) + 0.534 (TG/HDL-C) + 0.121 (METS-IR).

### 3.3 Model performance

**Figure 3:**
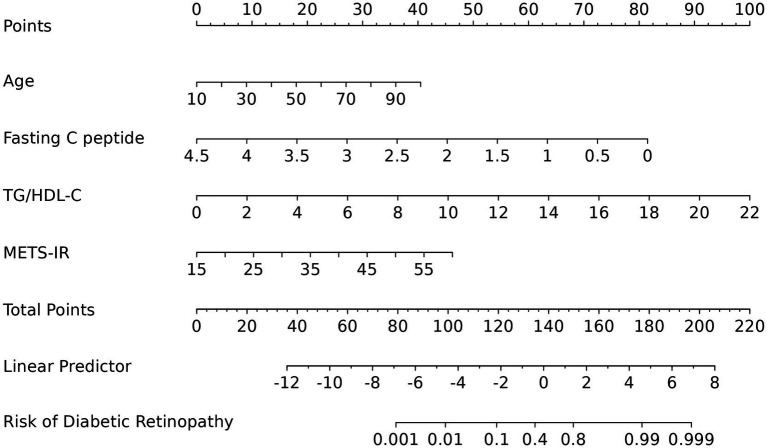
A nomogram for assessing DR risk in T2DM.

**Figure 4:**
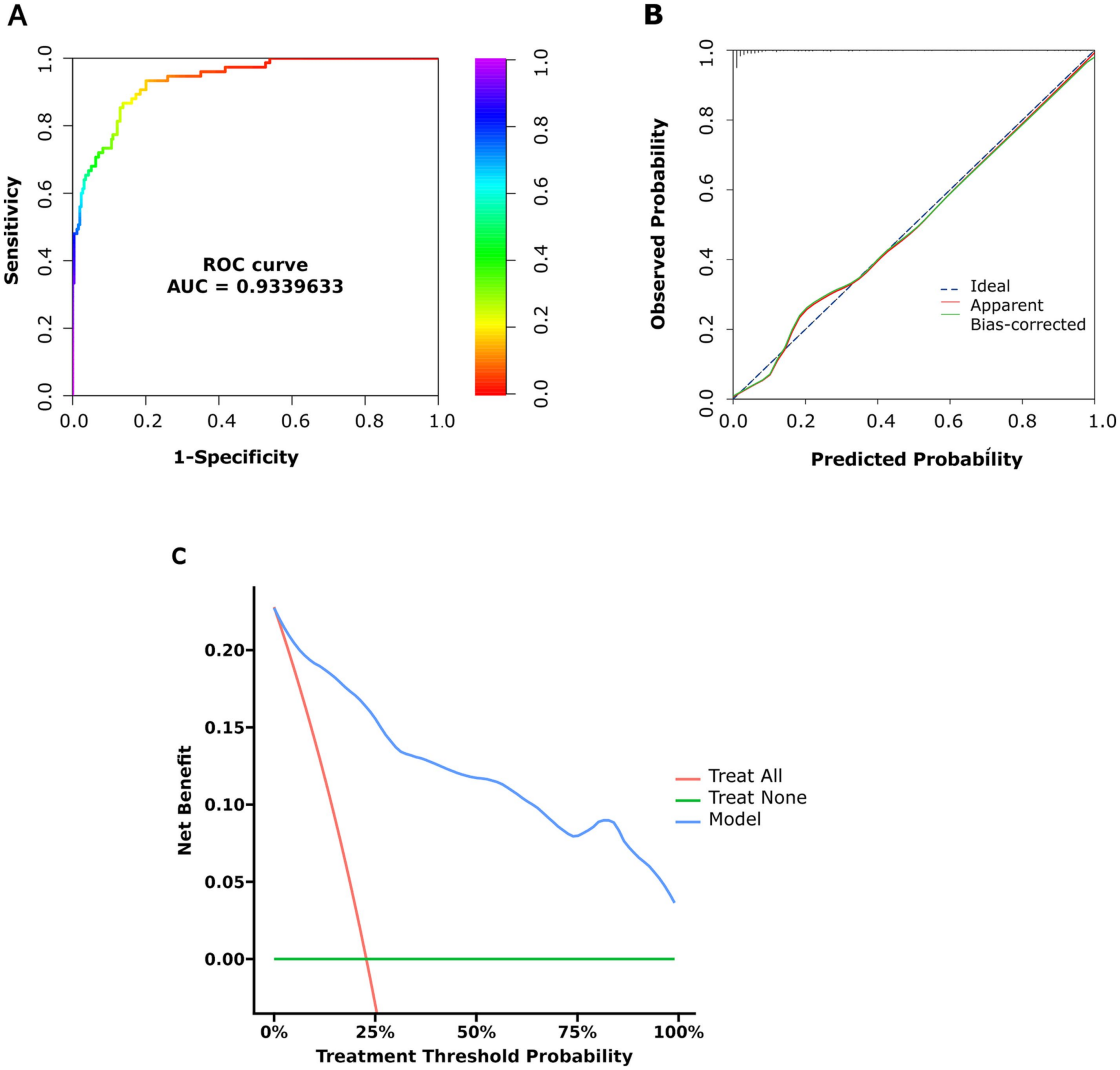
Validation of the nomogram model: **(A)** Calibration plot, **(B)** ROC curve, **(C)** DCA curve.

A nomogram incorporating the four predictors was constructed (Figure 3). The model achieved an AUC of 0.934 (95% CI 0.905–0.963), indicating excellent discrimination (Figure 4A). After bootstrap internal validation (1,000 resamples), the optimism-corrected C-index was 0.929 (95% CI 0.887–0.971). Calibration was satisfactory (intercept = 0, slope = 1, Brier score = 0.08), suggesting no systematic bias or overfitting (Figure 4B). Decision curve analysis demonstrated positive net benefit across a wide range of threshold probabilities, particularly within the clinically relevant 5–30% range for referral decisions (Figure 4C), supporting the model’s potential utility for risk stratification.

### 3.4 Baseline characteristics by DR severity

Among patients with DR, those with proliferative DR (PDR, *n* = 32) had significantly higher TG/HDL-C and METS-IR than those with non-proliferative DR (NPDR, *n* = 43) (*p* < 0.05). No other significant differences were observed (Table 4).

**Table 4:** Comparison of baseline characteristics between the NPDR and PDR groups.

Characteristic	Overall *N* = 75	NPDR *N* = 43	PDR *N* = 32	*p*-value
Sex, *n* (%)				0.443
Female	36 (48.0%)	19 (44.2%)	17 (53.1%)	
Male	39 (52.0%)	24 (55.8%)	15 (46.9%)	
Age (year)	60 (56, 67)	60 (54, 63)	62 (57, 72)	0.120
History of smoking				0.815
No	36 (48.0%)	19 (44.2%)	17 (53.1%)	
Yes	34 (45.3%)	21 (48.8%)	13 (40.6%)	
Once upon a time, now quit	5 (6.7%)	3 (7.0%)	2 (6.3%)	
History of drinking				0.436
No	34 (45.3%)	17 (39.5%)	17 (53.1%)	
Yes	37 (49.3%)	24 (55.8%)	13 (40.6%)	
Once upon a time, now quit	4 (5.3%)	2 (4.7%)	2 (6.3%)	
SBP (mmHg)	130 (123, 149)	126 (121, 148)	140 (129, 156)	0.071
DBP (mmHg)	83 (74, 94)	81 (74, 94)	83 (74, 94)	0.656
WBC (×10^9^/L)	6.70 (5.53, 8.63)	6.75 (5.56, 8.94)	6.44 (5.51, 8.62)	0.480
RBC (×10^12^/L)	4.47 (4.10, 4.76)	4.54 (4.19, 4.99)	4.38 (4.04, 4.67)	0.093
HGB (g/L)	137 (125, 145)	140 (126, 149)	133 (118, 142)	0.089
PLT (×10^9^/L)	222 (187, 259)	224 (187, 259)	221 (185, 255)	0.889
NEU (×10^9^/L)	4.50 (3.55, 6.48)	4.73 (3.70, 6.48)	4.38 (3.51, 6.40)	0.622
LYM (×10^9^/L)	1.50 (1.17, 1.90)	1.50 (1.20, 2.00)	1.38 (0.98, 1.90)	0.348
MON (×10^9^/L)	0.36 (0.26, 0.46)	0.36 (0.28, 0.46)	0.35 (0.25, 0.46)	0.431
PT (Sec)	12.70 (12.20, 13.30)	12.70 (12.40, 13.30)	12.40 (12.00, 13.50)	0.220
INR	1.05 (1.01, 1.10)	1.06 (1.03, 1.09)	1.05 (1.00, 1.13)	0.337
PTA (Sec)	90 (84, 94)	89 (85, 92)	90 (83, 95)	0.340
APTT (Sec)	26.6 (24.2, 29.2)	26.8 (24.5, 29.0)	26.2 (23.3, 29.3)	0.744
TT (Sec)	14.74 (13.39, 15.32)	14.33 (13.49, 15.02)	14.82 (13.21, 15.65)	0.567
Fib (g/L)	3.07 (2.68, 4.14)	3.00 (2.70, 3.75)	3.35 (2.39, 4.49)	0.541
HbA1c (%)	10.30 (8.70, 11.80)	10.30 (9.50, 13.00)	9.80 (8.15, 11.35)	0.063
FPG (mmol/L)	14 (10, 19)	14 (10, 19)	11 (9, 21)	0.510
TC (mmol/L)	5.24 (4.45, 6.11)	5.33 (4.60, 6.11)	4.88 (4.33, 6.16)	0.371
LDL-C (mmol/L)	3.05 (2.40, 3.59)	3.17 (2.50, 3.82)	2.84 (2.36, 3.34)	0.126
ALT (U/L)	17 (14, 21)	17 (13, 21)	17 (14, 22)	0.626
AST (U/L)	18 (16, 24)	18 (15, 23)	19 (17, 24)	0.258
TBIL (μmol/L)	12.6 (9.5, 15.0)	13.0 (9.8, 15.6)	10.7 (8.2, 14.1)	0.065
DBIL (μmol/L)	2.10 (1.70, 2.70)	2.20 (1.90, 2.80)	2.10 (1.60, 2.45)	0.233
BUN (mmol/L)	6.5 (4.8, 8.6)	6.0 (4.8, 7.8)	7.0 (5.0, 10.2)	0.076
CREA (μmol/L)	64 (50, 87)	66 (48, 87)	64 (54, 86)	0.645
UA (μmol/L)	294 (231, 353)	287 (259, 337)	316 (197, 386)	0.510
K (mmol/L)	4.11 (3.85, 4.45)	4.11 (3.95, 4.45)	4.03 (3.72, 4.47)	0.222
Na (mmol/L)	135.6 (133.6, 137.3)	135.0 (133.6, 136.5)	136.4 (133.0, 138.5)	0.232
CK (U/L)	68 (46, 88)	66 (48, 85)	71 (42, 103)	0.906
LDH (U/L)	153 (138, 195)	150 (134, 182)	156 (141, 202)	0.354
CK-MB (U/L)	15 (12, 19)	15 (12, 17)	16 (14, 20)	0.182
UACR (mg/g)	37 (16, 139)	37 (16, 104)	36 (15, 244)	0.649
0-h glucose (mmol/L)	7.82 (6.84, 9.17)	7.39 (6.78, 9.17)	8.02 (7.35, 9.05)	0.700
Fasting C peptide (ng/mL)	0.84 (0.49, 1.09)	0.78 (0.48, 1.09)	0.85 (0.50, 1.12)	0.692
BMI (kg/m^2^)	25.35 (23.81, 27.04)	24.97 (23.66, 26.37)	25.71 (24.05, 27.31)	0.108
TG/HDL-C	8.4 (4.6, 10.7)	6.8 (2.6, 8.4)	11.0 (10.3, 14.4)	<0.001
METS-IR	39.4 (36.6, 42.5)	37.0 (35.3, 39.3)	42.0 (39.9, 46.6)	<0.001

SBP, systolic blood pressure; DBP, diastolic blood pressure; WBC, white blood cell count; RBC, red blood cell; HGB, hemoglobin; PLT, platelet; NEU, neutrophils; LYM, lymphocytes; MON, monocytes; PT, prothrombin time; INR, international normalized ratio; PTA, prothrombin activity; APTT, activated partial thromboplastin time; TT, thrombin time; Fib, fibrinogen; HbA1c:hemoglobin A1c; FPG, fasting plasma glucose; TC, total cholesterol; LDL-C, low-density lipoprotein Cholesterol; ALT, alanine aminotransferase; AST, aspartate aminotransferase; TBil, total bilirubin; DBIL, direct Bilirubin; BUN, blood urea nitrogen; CREA, creatinine; UA, uric acid; K:kalium; Na, natrium; CK, creatine Kinase; LDH, lactate dehydrogenase; CK-MB, creatine kinase-myocardial band; UACR, urine albumin-to-creatinine ratio; 0-h glucose:0-h Glucose; BMI, body mass index; TG/HDL-C, triglyceride to high-density lipoprotein cholesterol ratio; METS-IR, metabolic syndrome insulin resistance index.

### 3.5 Exploratory analysis of factors associated with PDR

Univariate logistic regression identified TG/HDL-C (OR = 2.22 per unit, 95% CI 1.48–3.33, *p* < 0.001) and METS-IR (OR = 1.51 per unit, 1.24–1.84, *p* < 0.001) as positively associated with PDR, while HbA1c showed a negative association (OR = 0.79 per %, 0.64–0.99, *p* = 0.041). After Firth’s penalized logistic regression to correct for small-sample bias, both TG/HDL-C (OR = 3.10, 95% CI 1.77–7.21, *p* < 0.001) and METS-IR (OR = 1.80, 1.31–3.07, *p* < 0.001) remained independently associated with PDR (Table 5). These findings should be interpreted cautiously given the limited sample size (*n* = 32 for PDR).

**Table 5:** Univariate and Firth’s penalized logistic regression analysis of risk factors for PDR in T2DM.

Characteristic	Univariable			Firth’s penalized logistic regression		
	OR	95% CI	*p*-value	OR	95% CI	*p*-value
Sex, *n* (%)						
Female	—	—				
Male	0.70	0.28, 1.75	0.444			
Age (year)	1.04	0.99, 1.09	0.085			
History of smoking						
No	—	—	—			
Yes	0.69	0.27, 1.79	0.448			
Once upon a time, now quit	0.75	0.11, 5.01	0.762			
History of drinking						
No		—	—	—		
Yes	0.54	0.21, 1.40	0.207			
Once upon a time, now quit	1.00	0.13, 7.94	>0.999			
SBP (mmHg)	1.02	1.00, 1.04	0.125			
DBP (mmHg)	1.01	0.97, 1.04	0.753			
WBC (×10^9^/L)	0.93	0.77, 1.12	0.448			
RBC (×10^12^/L)	0.98	0.96, 1.01	0.138			
HGB (g/L)	0.62	0.31, 1.26	0.187			
PLT (×10^9^/L)	1.00	0.99, 1.01	0.852			
NEU (×10^9^/L)	0.96	0.84, 1.10	0.530			
LYM (×10^9^/L)	0.68	0.31, 1.49	0.331			
MON (×10^9^/L)	0.26	0.02, 3.20	0.291			
PT (Sec)	0.92	0.57, 1.48	0.733			
INR	0.34	0.00, 224.02	0.746			
PTA (Sec)	1.00	0.95, 1.05	0.986			
APTT (Sec)	1.03	0.95, 1.11	0.463			
TT (Sec)	1.14	0.94, 1.38	0.195			
Fib (g/L)	1.30	0.93, 1.81	0.121			
HbA1c (%)	0.79	0.64, 0.99	0.041	1.31	0.88,2.19	0.19
FPG (mmol/L)	0.99	0.92, 1.07	0.875			
TC (mmol/L)	0.85	0.57, 1.28	0.436			
LDL-C (mmol/L)	0.65	0.36, 1.17	0.151			
ALT (U/L)	0.98	0.95, 1.02	0.326			
AST (U/L)	1.00	0.96, 1.04	0.929			
TBIL (μmol/L)	0.93	0.84, 1.02	0.131			
DBIL (μmol/L)	0.80	0.52, 1.24	0.323			
BUN (mmol/L)	1.13	0.99, 1.28	0.068			
CREA (μmol/L)	1.00	0.99, 1.01	0.569			
UA (μmol/L)	1.00	1.00, 1.01	0.578			
K (mmol/L)	0.46	0.17, 1.26	0.132			
Na (mmol/L)	1.05	0.92, 1.20	0.444			
CK (U/L)	1.00	1.00, 1.01	0.333			
LDH (U/L)	1.01	0.99, 1.02	0.274			
CK-MB (U/L)	0.99	0.96, 1.02	0.597			
UACR (mg/g)	1.00	1.00, 1.00	0.570			
0-h glucose (mmol/L)	1.00	0.79, 1.26	0.974			
Fasting C peptide (ng/mL)	1.20	0.44, 3.31	0.725			
BMI (kg/m^2^)	1.19	0.97, 1.46	0.088			
TG/HDL-C	2.22	1.48, 3.33	<0.001	3.10	1.77, 7.21	<0.001
METS-IR	1.51	1.24, 1.84	<0.001	1.80	1.31, 3.07	<0.001

WBC, white blood cell count; RBC, red blood cell; HGB, hemoglobin; PLT, platelet; NEU, neutrophils; LYM, lymphocytes; MON, monocytes; PT, prothrombin time; INR, international normalized ratio; PTA, prothrombin activity; APTT, activated partial thromboplastin time; TT, thrombin time; Fib, fibrinogen; HbA1c:hemoglobin A1c; FPG, fasting plasma glucose; TC, total cholesterol; TG, triglycerides; HDL-C, High-Density Lipoprotein Cholesterol; LDL-C, low-density lipoprotein Cholesterol; ALT, alanine aminotransferase; AST, aspartate aminotransferase; TBIL, total bilirubin; DBIL, direct Bilirubin; BUN, blood urea nitrogen; CREA, creatinine; UA, uric acid; K:kalium; Na, natrium; CK, creatine Kinase; LDH, lactate dehydrogenase; CK-MB, creatine kinase-myocardial band; UACR, urine albumin-to-creatinine ratio; 0-h glucose:0-h Glucose; BMI, body mass index; TG/HDL-C, triglyceride to high-density lipoprotein cholesterol ratio; METS-IR, metabolic syndrome insulin resistance index.

## 4 Discussion

We investigated the association of TG/HDL-C and METS-IR with DR in T2DM, aiming to identify factors independently associated with DR, evaluate their utility for risk stratification, and explore their relationship with disease severity. The main findings are as follows: (1) Compared with patients without DR, those with DR were older and had higher BMI, PLT, Fib, Gluc, LDH, TG/HDL-C and METS-IR, but lower RBC, HGB, ALT, AST and fasting C-peptide. (2) LASSO and logistic regression identified age, TG/HDL-C and METS-IR as factors positively associated with DR, whereas fasting C-peptide showed an inverse association. (3) The model incorporating these factors achieved an AUC of 0.934 (95% CI 0.905–0.963) and an optimism-corrected C-index of 0.929 (0.887–0.971) after bootstrap validation. Calibration was satisfactory, and decision curve analysis indicated positive net benefit. (4) Exploratory analysis of DR severity using Firth’s penalized logistic regression showed that TG/HDL-C and METS-IR were independently associated with PDR.

Patients with T2DM and DR were older and exhibited poorer glycemic control, coagulation abnormalities (elevated PLT and Fib) and higher TG/HDL-C levels (Table 1). An age–period–cohort analysis based on Chinese data (1990–2021) reported that DR prevalence increases with age, peaking in the 40–60 year range (18). As a chronic complication of diabetes, DR typically takes approximately 8 years to progress to mild stages, after which further progression to moderate, severe or proliferative DR may occur within 1–2 years (19). This is thought to reflect microvascular pathology including microaneurysms, increased vascular permeability, exudates and intraretinal hemorrhages. Mechanistically, hyperglycemia may promote retinal microthrombus formation through multiple pathways: disrupting coagulation and fibrinolysis (20), damaging endothelium and activating platelets (21), and upregulating vascular endothelial growth factor (VEGF), which increases fibrinogen and blood viscosity (22).

Triglycerides may penetrate the damaged retinal microvascular barrier, deposit as hard exudates and, together with hyperglycaemia and upregulated VEGF, drive macular oedema and microvascular occlusion, leading to progressive visual impairment (23). A retrospective study of 110 patients with T2DM in Korea reported that triglyceride levels were associated with the presence and severity of diabetic macular oedema (24), supporting a synergistic role of lipid and glucose dysregulation in DR progression and suggesting that triglyceride control may be important for delaying DR, particularly macular oedema. In addition, fluctuating hyperglycaemia can activate protein kinase C and oxidative stress, promote endothelial apoptosis and increase LDH release (25). A study of 3,476 patients with diabetes found that LDH levels above 134 U/L were associated with significantly higher DR risk (26), further supporting LDH as a marker of endothelial injury in DR pathogenesis.

TG/HDL-C and METS-IR are commonly considered markers of insulin resistance and risk factors for T2DM and its microvascular complications. Exclusion of patients on lipid-lowering therapy ensured that the observed elevations in TG/HDL-C and METS-IR reflect intrinsic metabolic dysregulation associated with DR, rather than pharmacological modulation. A study of 979 white patients with T2DM reported that TG/HDL-C was a risk factor for DR (27), and a cross-sectional study of 2,211 patients with diabetes found positive associations of METS-IR and TG/HDL-C with DR (28), consistent with our finding that both indices were independently associated with DR in multivariable analysis. We also observed an inverse association between fasting C-peptide and DR (OR = 0.12 per ng/mL, 95% CI 0.05–0.27; Table 3), suggesting a potential protective effect. A study of 130 patients with T2DM similarly reported that higher C-peptide levels were associated with lower risk of DR, diabetic peripheral neuropathy and diabetic nephropathy, as well as better glycaemic control (HbA1c < 7.5%) (29). Although this may seem counter to the notion that hyperinsulinaemia is harmful, it points to a more fundamental clinical reality: preserved pancreatic *β*-cell function is key to protecting against microvascular complications. In T2DM, patients with insulin resistance but maintained β-cell compensatory capacity (that is, those capable of substantial insulin and C-peptide secretion) may have better retinal protection. Mechanistically, C-peptide has been shown to reduce hyperglycaemia-induced upregulation of vascular cell adhesion molecule-1 (VCAM-1), interleukin-8 (IL-8) and macrophage chemoattractant protein-1 (MCP-1), decreasing monocyte adhesion to endothelial cells (30, 31). It also prevents endothelial apoptosis by modulating caspase-3 expression and increasing levels of the anti-apoptotic molecule Bcl-232. Additional studies suggest that C-peptide reduces reactive oxygen species (ROS) production through multiple targets, including NADPH oxidase (32), AMPK-*α* (33) and transglutaminase 2 (34), thereby mitigating endothelial damage.

The model incorporating age, fasting C-peptide, TG/HDL-C and METS-IR showed good discrimination for DR, with an AUC of 0.934 and an optimism-corrected C-index of 0.929. Calibration was satisfactory, and decision curve analysis indicated positive net benefit. These findings suggest that DR risk in T2DM is associated not with a single factor but with the combined effects of insulin resistance (reflected by METS-IR), lipid dysregulation (reflected by TG/HDL-C) and *β*-cell function (reflected by fasting C-peptide). For patients with T2DM of 5–10 years duration, some may not undergo regular fundus examinations, leading to missed DR diagnosis. The present model, based on routinely available clinical measures, offers a practical tool for early identification of hospitalized patients at high risk of DR. Although it cannot replace ophthalmologic examination, it may facilitate risk stratification and inform comprehensive management targeting multiple metabolic factors beyond glycaemic control alone.

We further stratified the 75 patients with NPDR and PDR groups. In univariate analysis, HbA1c showed a protective trend, but this was not retained in Firth’s penalized regression. By contrast, TG/HDL-C (OR = 3.10, 95% CI 1.77–7.21) and METS-IR (OR = 1.80, 1.31–3.07) remained independently associated with PDR. These findings suggest that progression from NPDR to PDR is associated with severe lipid dysregulation (elevated TG/HDL-C) and systemic insulin resistance (elevated METS-IR), even in patients with well-controlled glycaemia. In patients with DR, lipotoxicity resulting from severe lipid dysregulation may contribute to retinal endothelial injury and pathological neovascularization (35). The interplay between insulin resistance and TG/HDL-C could create a vicious cycle that perpetuates microvascular damage, accelerating the transition from NPDR to PDR. Given the small sample size of the PDR subgroup (*n* = 32), these results are exploratory and warrant confirmation in studies with larger numbers of events.

## 5 Limitations

This study has several limitations. First, the data were derived from a single Asian cohort; given known differences in genetic background, lifestyle and dietary habits across populations, the generalizability of our findings to other groups requires further external validation. Second, the modest sample size may limit the robustness of statistical models and the precision of estimates, this validation is internal only, and that the model’s performance may be optimistic. Third, despite the use of Firth’s penalized regression, residual confounding from unmeasured factors—such as diet, physical activity, genetic background, comorbidities and use of glucose-lowering medications—cannot be excluded. The exclusion of patients on lipid-lowering therapy within 3 months prior to admission, while minimizing interference with lipid measurements, may have introduced selection bias; future studies should collect detailed medication histories and adjust for this potential confounder. Importantly, the study population comprised hospitalized patients with T2DM of 5–10 years’ duration receiving intensive insulin therapy. These individuals likely have more severe metabolic disturbances than outpatients with milder disease, and caution is warranted when extrapolating our conclusions to primary care or general diabetes clinic populations. Larger, multicentre cohort studies encompassing diverse demographic and clinical characteristics are needed to validate the utility and predictive performance of TG/HDL-C and METS-IR across different healthcare settings.

## 6 Conclusion

In hospitalized patients with T2DM receiving insulin therapy, TG/HDL-C and METS-IR were independently associated with diabetic retinopathy. A prediction model incorporating these two indicators showed good discrimination and calibration in this population, with decision curve analysis suggesting potential utility for risk stratification. Exploratory analysis of proliferative diabetic retinopathy indicated possible associations with disease progression, although these findings require validation in larger studies.

## Data Availability

The raw data supporting the conclusions of this article will be made available by the authors, without undue reservation.
